# N6-methyladenosine-mediated CELF2 regulates CD44 alternative splicing affecting tumorigenesis via ERAD pathway in pancreatic cancer

**DOI:** 10.1186/s13578-022-00844-0

**Published:** 2022-08-08

**Authors:** Shihui Lai, Yan Wang, Ting Li, Yihong Dong, Yihao Lin, Liang Wang, Shangeng Weng, Xiang Zhang, Chengjie Lin

**Affiliations:** 1 Department of Hepatopancreatobiliary Surgery, The First Affiliated Hospital of Fujian Medical University, Fuzhou, 350001 Fujian China; 2 Department of Oncology, Fujian Provincial Hospital, Provincial Clinical College of Fujian Medical University, Fuzhou, 350001 Fujian China

**Keywords:** Pancreatic cancer (PC), Alternative splicing, N6-methyladenosine (m6A), CELF2, ERAD pathway

## Abstract

**Background:**

Alternative splicing (AS) of genes has been found to affect gene stability, and its abnormal regulation can lead to tumorigenesis. CELF2 is a vital splicing factor to participate in mRNA alternative splicing. Its downregulation has been confirmed to promote the occurrence and development of pancreatic cancer (PC). However, the regulatory role and mechanisms in PC has not been elucidated.

**Results:**

CELF2 was downregulated in PC tissues, which affected tumor TNM stage and tumor size, and low expression of CELF2 indicated a poor prognosis of PC. In vivo and in vitro experiments showed that abnormal expression of CELF2 affected the stemness, apoptosis, and proliferation of PC cells. Furthmore, we also found that CELF2 was targeted by ALKBH5 for m6A modification, leading to CELF2 degradation by YTHDF2. Bioinformatic analysis of AS model based on the TCGA database indicated that CELF2 could target CD44 to form different spliceosomes, thereby affecting the biological behavior of PC cells. The conversion of CD44s to CD44V is the key to tumorigenesis. Transcriptomic analysis was conducted to reveal the mechanism of CELF2-mediated CD44 AS in PC. We found that CELF2-mediated splicing of CD44 led to changes in the level of endoplasmic reticulum stress, further regulating the endoplasmic reticulum-associated degradation (ERAD) signaling pathway, thereby affecting apoptosis and cell stemness. In addition, ERAD signaling pathway inhibitor, EerI, could effectively reverse the effect of CD44 on tumors.

**Conclusions:**

This study indicates that N6-methyladenosine-mediated CELF2 promotes AS of CD44, affecting the ERAD pathway and regulating the biological behavior of PC cells. CELF2 is expected to be a new target for targeted-drug development.

**Supplementary Information:**

The online version contains supplementary material available at 10.1186/s13578-022-00844-0.

## Background

Pancreatic cancer (PC) is one of the most fatal tumors, with a 5-year survival rate of only 8%. The number of newly diagnosed PC cases in China accounts for 19.45% of the global total, and the number of deaths accounts for 19.27%, ranking among the top in the world [1]. PC is a hidden, aggressive, and rapidly developing tumor. Most of the patients have lost the opportunity for operation. Even if it can be treated surgically, the incidence of postoperative recurrence and metastasis is also high [2]. Therefore, in-depth understanding of the mechanisms involved in the progression of PC contributes to the development of new effective targets for the treatment of this fatal disease. The exploration of new strategies to improve the prognosis of patients has become the forefront of PC treatment research.

In recent years, studies have found that alternative splicing (AS) plays an important role in tumorigenesis [3]. AS is defined as splicing pre-mRNA from a common precursor to generate multiple transcripts, and its study may provide a new potential research field, which is meaningful to improve the diagnosis, prediction, and even the development of new therapeutic drugs. AS can affect gene stability, and its abnormal regulation can lead to disease [4, 5]. Studies have shown that AS has a significant impact on tumor biology [6]. Therefore, screening of PC-related AS genes and in-depth exploration of the AS mechanisms may provide an important framework for effective clinical treatment of patients with PC.

Cytidine-uridine-guanosine-binding protein 2 (CUGBP) Elav-like family member 2 (CELF2) is a member of the CELF protein family. CELF2 is an RNA-binding protein that can be used as a splicing factor to participate in mRNA splicing, editing, and translation regulation [7]. CELF2 downregulation has been confirmed to promote the occurrence and development of gastric malignant tumors [8]. However, there are few studies on the role of CELF2 in PC. One study has shown that CELF2 was downregulated and affected the sensitivity to chemotherapeutics in PC [9]. The role of CELF2 as a splicing factor in cancer has not yet been elucidated. In this study, we used clinical samples, cytology, animal experiments, and high-throughput sequencing to further explore the effects of AS of CELF2 regulatory genes on the biological behavior of PC cells, aiming to provide new ideas for the treatment of PC.

## Results

### CELF2 expression is low in pancreatic cancer and affects the prognosis of patients

**Fig. 1:**
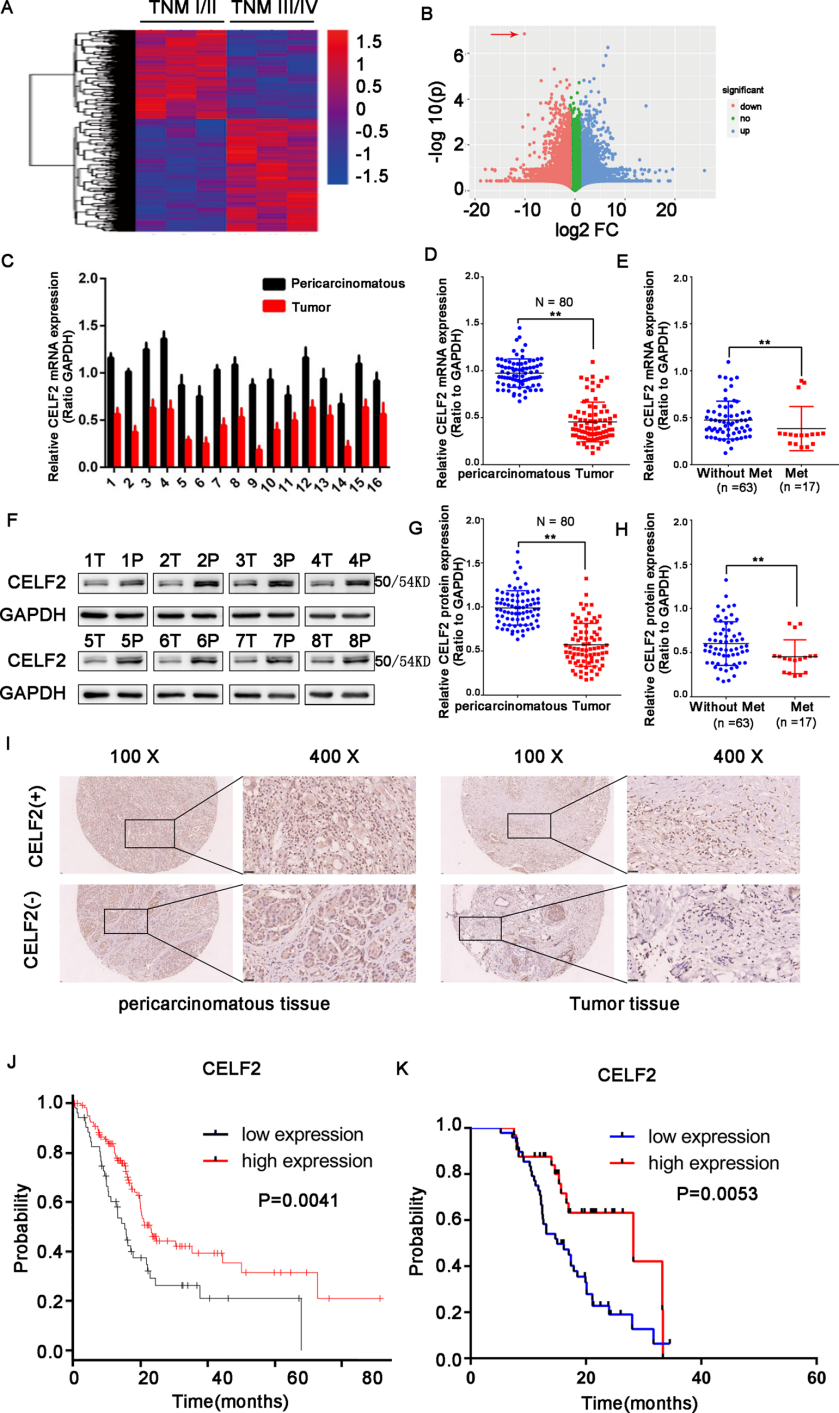
High CELF2 expression in patient samples was correlated with longer overall survival. **A**, **B** Transcriptional sequencing was conducted to analyze differential expression genes in different TNM stages of PC. **A** The heapmap of the differential expression genes. **B** The volcano plot of the differential expression genes. The red arrow presented the expression of CELF2. **C** The mRNA expression of *CELF2* in PC tissues and pericarcinomatous tissue was analyzed by qRT-PCR. **D** The mRNA expression of *CELF2* in tumor tissues was lower than that in pericarcinomatous tissue. **E** The lower expression of *CELF2* mRNA is closely related to the distant metastasis of tumors. **F** Western blot was used to analyze the protein expression of CELF2 in tumor (T) and pericarcinomatous tissue (P). **G** CELF2 protein expression was significantly lower in tumor tissues than that in pericarcinomatous tissue. **H** The lower expression of CELF2 protein is closely related to the distant metastasis of tumors. **I** CELF2 was overexpressed in the nucleus of PC tissues, but its expression was low in paired pericarcinomatous tissue. The label "CELF2(+)" represents positive expression and "CELF2(−)" represents negative expression in pancreatic cancer cells. **J** The survival of pancreatic cancer patients with high CELF2 expression was significantly better than that of patients with low expression from the TCGA database. **K** Kaplan–Meier analysis revealed that overall survival in the high CELF2 expression group was better than that in low expression groups in our clinical center. *P < 0.05, **P < 0.01

First, transcriptional sequencing was conducted to analyze differential expression genes in different TNM stages of PC, the results suggested that the higher the TNM stage, the lower expression of CELF2, which also revealed that CELF2 played a key role in the progression of PC (Fig. 1A, B). To confirm the relationship between the expression level of CELF2 and prognosis of PC, CELF2 expression levels in PC tissues and pericarcinomatous tissue were examined by western blot and RT-PCR. As shown in Fig. 1C, D, F, and G, the protein and mRNA expression levels of CELF2 were significantly lower in PC tissues than in pericarcinomatous tissue, and low CELF2 expression is closely related to the occurrence of distant tumor metastasis (Fig. 1E and H). In the IHC assay, CELF2 was found to be mainly expressed in the nucleus, and CELF2 expression in tumor tissues was also significantly lower than that in pericarcinomatous tissue (Fig. 1I). We further analyzed the PC data in the TCGA database, and we found that the survival time of patients with low CELF2 expression was significantly shorter than that of patients with high CELF2 expression (Fig. 1J). This result is consistent with the results of our center (Fig. 1K). Combined with clinicopathological characteristics, we found that abnormal expression of CELF2 is closely related to tumor size, lymph node metastasis, and distant metastasis, but not to age, gender, CEA, or CA199 (Table 1).

### ALKBH5-mediated m6A demethylation is involved in CELF2 downregulation

**Fig. 2:**
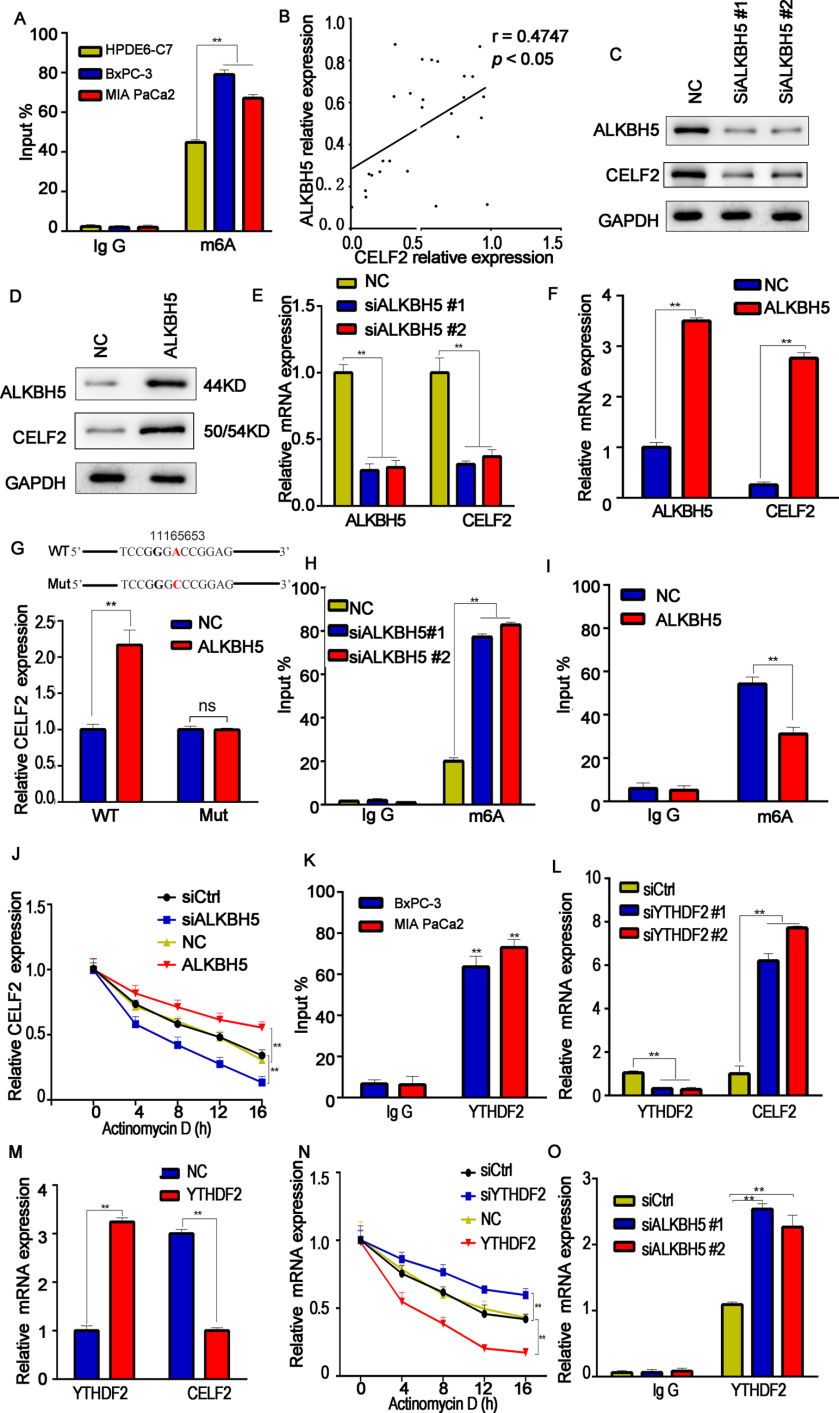
ALKBH5-mediated m6A modification is involved in the low expression of CELF2. **A** The RIP qRT-PCR assay showed the enrichment of m6A-modified CELF2 in PC cells (BxPC-3 and MIA PaCa2) compared with the normal pancreatic cell line HPDE6-C7. **B** The positive correlation between the levels of ALKBH5 and CELF2 in pancreatic cancer tissue was analyzed by RT-PCR. **C**–**F** The effect of ALKBH5 silencing or overexpression on CELF2 expression. **G** The m6A modification locus 1116565 of CELF2 can be modified by ALKBH5 predicted by the online website http://m6avar.renlab.org/, which was confirmed by the following luciferase reporter assay. **H** and **I** RIP qRT-PCR results showing the enrichment of m6A-modified CELF2 after ALKBH5 silencing or overexpression. **J** CELF2 stability analysis in BxPC-3 cells with CELF2 silencing or overexpression in the presence of actinomycin D. *P < 0.05, **P < 0.01. **K** The enrichment of the YTHDF2 on CELF2 in PC cells was analyzed by RIP-RT-qPCR assay. **L**, **M** Effect of YTHDF2 knockdown or overexpression on CELF2 expression. **N** Actinomycin D assay results showed the effect of YTHDF2 knockdown or overexpression on CELF2 stability. **O** RIP-RT-qPCR assay results show the enrichment of YTHDF2 on CELF2 while ALKBH5 silencing. Data are shown as the mean SD of three replicates. *P < 0.05; **P < 0.01

m6A modification has been shown to regulate RNA stability [12]. Many m6A sites were found within CELF2 in the RMBase database (http://rna.sysu.edu.cn/rmbase/index.php). Therefore, we hypothesized that CELF2 might be downregulated through changes in its N6-methyladenosine (m6A) modification levels. m6A RNA immunoprecipitation (RIP) and qRT-PCR results showed that m6A levels in PC cell lines (BxPC-3 and MIA PaCa2) were significantly higher than in normal pancreatic cells (Fig. 2A). ALKBH5 is a demethylase, which has been proved to induce gene demethylation to affect mRNA stability, resulting in abnormal gene expression. We further detected the mRNA expression levels of ALKBH5 and CELF2 in pancreatic cancer tissues, and found that the CELF2 expression level is positively correlated with the ALKBH5 expression level (Fig. 2B). ALKBH5 expression level in pancreatic cancer tissue and cancer cell lines were shown in Additional file 1: Figure S1A. ALKBH5 siliencing significantly decreased CELF2 levels (Fig. 2C, E), whereas ALKB5 overexpression increased CELF2 levels (Fig. 2D, F). Here, we further predicted the m6A modification sites of CELF2 through the online website http://m6avar.renlab.org/. The preliminary experimental verification indicated that the site 1116565 can be modified by ALKBH5. In the luciferase assay, ALKBH5 overexpression resulted in the increase of luciferase activity in the wild-type CELF2 gourp, however, No change of luciferase activity was observed in the mutant-type CELF2 group (Fig. 2G). Me-RIP assays showed that ALKBH5 silencing increased the m6A modification of CELF2 in PC cells (Fig. 2H), while overexpression of ALKBH5 led to the opposite result (Fig. 2I). In the presence of actinomycin D (a drug that blocks the de novo RNA synthesis), ALKBH5 depletion decreased the stability of CELF2 (Fig. 2J). Overall, these data indicated that ALKBH5 acted as an m6A erasers and maintains low CELF2 expression in PC cells.

YT51-B homology omain family 2 (YTHDF2), a essential “reader” of m6A, maintains the stability of m6A-modified mRNAs [13]. The expression of YTHDF2 was higher in pancreatic cancer tissue than that in precarcinomatous tissue (Additional file 1: Figure S1B). The direct binding between YTHDF2 and CELF2 was confirmed by RIP assay (Fig. 2K). In addition, CELF2 was up-regulated while silencing YTHDF2 in BxPC-3, whereas it was downregulated while YTHDF2 overexpression (Fig. 2L-M). In the actinomycin D assay, YTHDF2 overexpression increases degradation of CELF2 gene expression (Fig. 2N). Furthermore, the expression of YTHDF2 was significantly up-regulated when ALKBH5 was silienced by RIP assay (Fig. 2O). All results above indicated that the "eraser" ALKBH5 might cause CELF2 m6A demethylation, thereby affecting CELF2 expression mediated by the "reader" YTHDF2 in PC cells.

### Abnormal expression of CELF2 affects the proliferation, invasion, and migration of pancreatic cancer cells

**Fig. 3:**
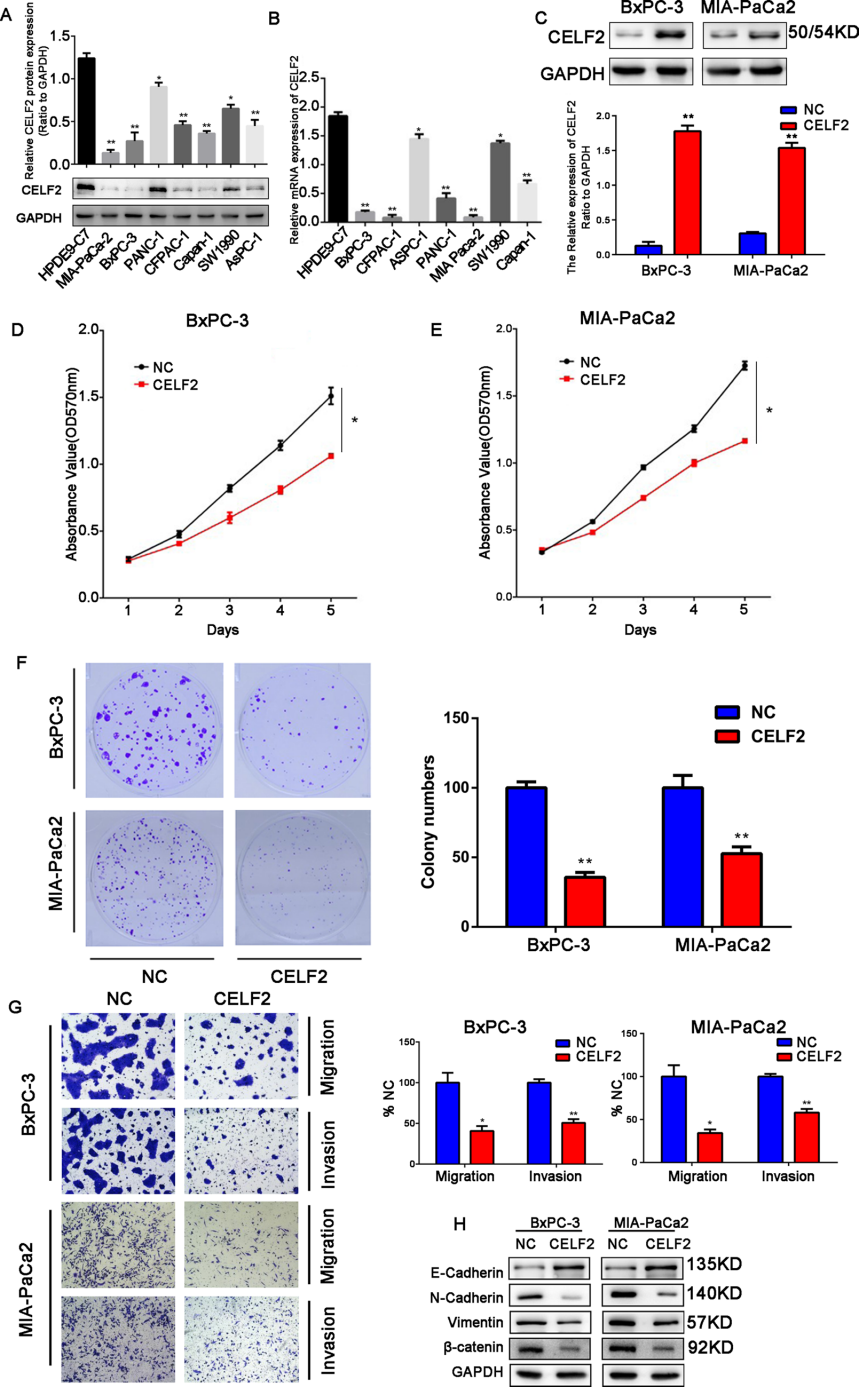
CELF2 upregulation inhibited the proliferation, migration, and invasion of PC cells. **A** and **B** Western blot (**A**) and qRT-PCR (**B**) analyses were used to measure the protein and mRNA levels of CELF2 in various PC cell lines and normal pancreatic cells. **C** Western blot was used to measure the protein levels of CELF2 in BxPC-3 and MIA PaCa2 cells 48 h after transfection with a CELF2 overexpression plasmid. **D** and **E** The CCK-8 assay revealed CELF2 overexpression inhibited the proliferation of BxPC-3 (**D**) and MIA PaCa2 cells (**E**). **F** CELF2 overexpression inhibited colony formation of BxPC-3 and MIA PaCa2 cells in vitro. **G** Transwell analysis revealed that the invasion and migration of BxPC-3 and MIA PaCa2 cells were significantly decreased when CELF2 was overexpressed. **H** EMT markers in cells with high expression levels of CELF2 were detected by western blot. Data are shown as the mean ± SD of three replicates. *P < 0.05, **P < 0.01

To further explore the potential biological functions of CELF2 in PC cells, the expression levels of CELF2 in one normal pancreatic cell line (HPDE6-C7) and seven PC cell lines were measured. We found that the protein and mRNA levels of CELF2 in tumor cells were lower than in normal pancreatic cells (Fig. 3A and 3B). Therefore, we selected two PC cell lines with relatively low CELF2 expression (BxPC-3 and MIA PaCa2) for follow-up experiments. CELF2 overexpression plasmids and NCs were transfected into BxPC-3 and MIA PaCa2 cells to explore the effects of CELF2 on PC cells, CELF2 was significantly upregulated after transfection (Fig. 3C). Compared with the NC group, the proliferation and cloning ability of BxPC-3 and MIA PaCa2 cells overexpressing CELF2 were significantly reduced (Fig. 3D–F). The Transwell assay suggested that the invasion and migration ability of PC cells is significantly reduced after CELF2 overexpression (Fig. 3G). Epithelial–mesenchymal transition (EMT) is considered one of the key mechanisms affecting the invasion and migration of PC cells. Therefore, we further studied the effect of CELF2 on EMT. The results showed that the expression level of the epithelial marker E-cadherin was increased and the expression levels of the mesenchymal markers N-cadherin, vimentin, and β-catenin were significantly decreased after CELF2 overexpression (Fig. 3H).

### Abnormal CELF2 affects the stemness and apoptosis of pancreatic cancer cells

**Fig. 4:**
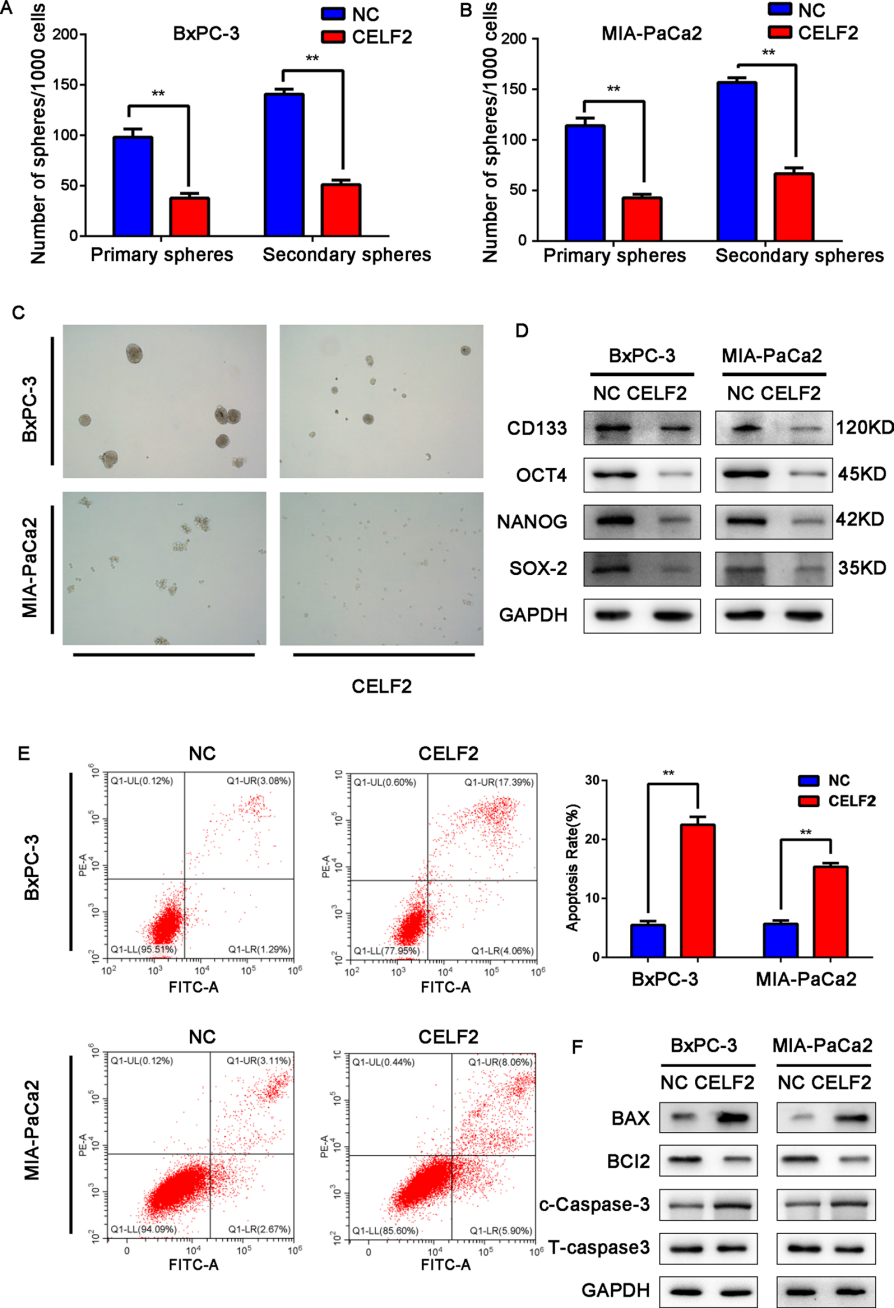
CELF2 downregulation inhibits apoptosis and maintains the stemness of PC cells. **A**–**C** Representative images of sphere formation induced by CELF2 overexpression in BxPC-3 and MIA PaCa2 cells. The surviving colonies were measured for the number of tumorspheres. **D** The expression levels of PC cell stemness markers, including CD133, OCT4, NANOG, and SOX-2, were examined by western blot. **E** Flow cytometry was used to assess apoptosis of PC cells in the CELF2 overexpression and NC groups. **F** The expression levels of apoptosis markers, including BAX, BCL-2, and caspase-3, were examined by western blot. Data are shown as the mean ± SD of three replicates. *P < 0.05, **P < 0.01

Sphere formation and holoclone assays were conducted to further explore whether CELF2 affects the cell stemness of PC cells. We found that the spheroidizing ability of PC cells was significantly inhibited when CELF2 was overexpressed in BxPC-3 and MIA PaCa2 cells (Fig. 4A–C). We further measured the expression levels of the pancreatic stem cell-like cell markers CD133, OCT4, NANOG, and SOX-2, and we found that these markers were significantly downregulated when CELF2 was overexpressed compared with the control group (Fig. 4D). This is consistent with the results of cell phenotyping experiments.

To further explore the mechanisms by which CELF2 affects the biological behavior of PC cells, we measured the apoptosis rate of cells overexpressing CELF2 by flow cytometry. The results showed that CELF2 significantly promoted apoptosis (Fig. 4E). The expression levels of apoptosis-related proteins were further detected by western blot. Compared with the control group, the expression of BCL-2 in the CELF2 overexpression group was significantly decreased, and the expression of caspase-3 and BAX were significantly increased (Fig. 4F).

### CELF2 inhibits tumor growth and distant metastasis in vivo

**Fig. 5:**
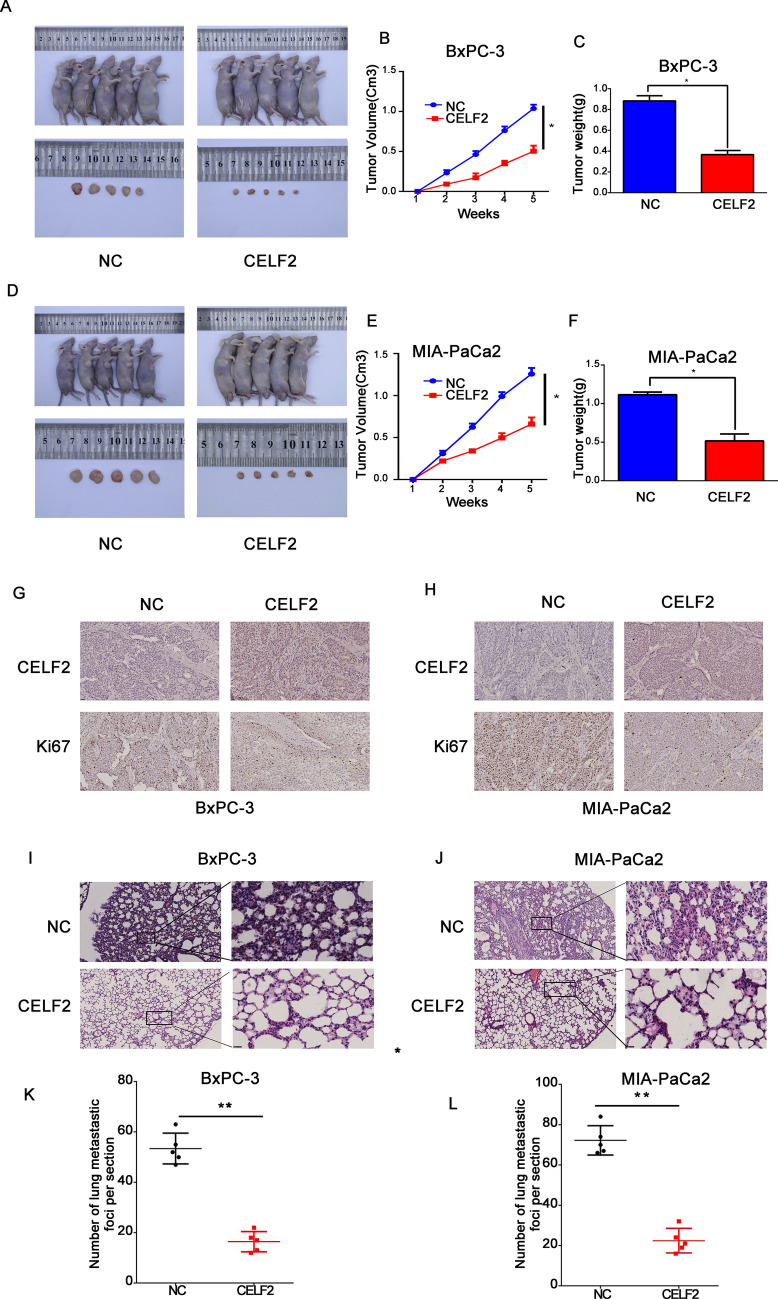
CELF2 inhibited the formation and lung metastasis of tumors in vivo. **A**–**F** CELF2 overexpression inhibited tumor formation in vivo*.* **A** and **D** BxPC-3 and MIA PaCa2 cells stably transfected with CELF2 overexpression plasmid or empty vector were injected in the flank of nude mice, which were sacrificed 5 weeks after injection. Then **B** and **E** tumor weight and **C** and **F** tumor volume were measured. **G** and **H** Ki-67 expression in the tumors of CELF2 overexpression and NC groups. **I**–**L** CELF2 overexpression inhibited lung metastasis in vivo. BxPC-3 and MIA PaCa2 cells stably transfected with CELF2 overexpression plasmid or empty vector were injected via the tail vein, and 5 weeks after injection the mice were sacrificed. **I** and **J** H&E staining of lung metastases. **K** and **L** Statistical analysis of the number of lung metastatic foci per section after CELF2 overexpression. CELF2 overexpression inhibited lung metastasis of PC cells. The data are expressed as mean ± SD of three replicates. *P < 0.05, **P < 0.01

The effect of CELF2 on tumor growth and distant metastasis was further explored in vivo. First, we performed the subcutaneous tumor formation experiment by injecting the cells that were transduced using the viruses and stably express the control vector or CELF2 into nude mice. The tumor volume and weight of the nude mice in the CELF2 overexpression group were significantly lower than those in the control group after 5 weeks (Fig. 5A–F). In addition, the expression level of Ki-67 in the tumor tissues of the CELF2 overexpression group was significantly lower than that of the control group (Fig. 5G and H), which further confirmed the anti-tumor effect of CELF2. Cells that were transduced with CELF2 overexpression and NC lentiviruses were injected through the tail vein to explore their effects on distant metastasis. The results showed that CELF2 could inhibit lung implantation of PC cells (Fig. 5I–L). All of the above data supported the cancer-inhibiting role of CELF2 in PC development.

### CELF2 makes CD44 form different spliceosomes to exert anti-tumor effects

**Fig. 6:**
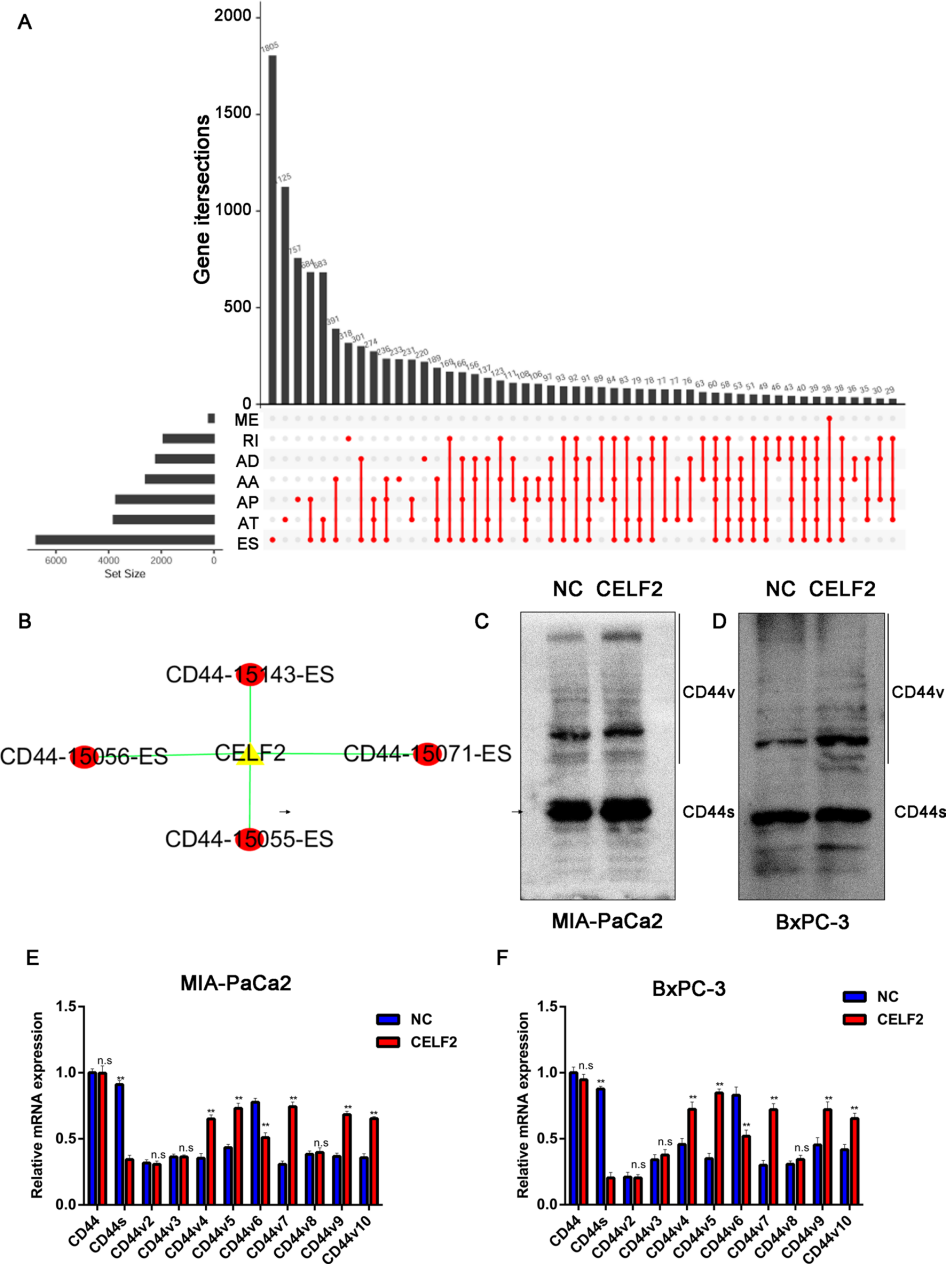
CELF2 made CD44 form different spliceosomes to exert anti-tumor effects. **A** An alternative splicing model with the alternative splicing genes involved in pancreatic cancer based on the TCGA database was established. **B** CELF2-mediated CD44 performed alternative splicing. **C** and **D** Protein levels of the different spliceosomes of CD44 after CELF2 overexpression were analyzed by western blot. **E** and **F** The different CD44 spliceosomes were detected after CELF2 overexpression in BxPC-3 and MIA PaCa2 cells by RT-PCR. Compared with the control group, the expression of CD44s was significantly decreased after overexpression of CELF2, while the expression levels of nine other spliceosomes, but not CD44v6, were increased. The results are consistent with the observed changes in CD44 mRNA spliceosome levels. Abbreviations in Additional file 1: Figure S1A: *AA* alternate acceptor, *AD* alternate donor site, *AP* alternate promoter, *AT* alternate terminator, *ES* exon skip, *ME* mutually exclusive exons, *RI* retained intron. The data are expressed as mean ± SD of three replicates. *P < 0.05, **P < 0.01

CELF2 is an important AS factor, which plays a key role in tumorigenesis by participating in AS [14]. Studies have shown that the expression of CELF2 in PC is downregulated and affects the sensitivity to chemotherapy drugs [9]. However, the role of CELF2 as a splicing factor in PC and the underlying mechanisms remain unclear. By establishing an AS model with the AS genes involved in PC based on the TCGA database, we found that there were a large number of AS behaviors in PC (Fig. 6A). In addition, CD44 could be spliced by CELF2, and formed different isoforms through exon skip (ES) (Fig. 6B). To further confirm these results, the different isoforms of CD44 were detected in HPDE9-C7 (Additional file 1: Figure S1C) and BxPC-3 / MIA PaCa2 cells overexpressing CELF2 by RT-PCR and Western-blot. Compared with the control group, the expression of CD44s was significantly decreased after overexpression of CELF2, while the expression levels of other spliceosomes were increased (Fig. 6C and D). Subsequently, we measured mRNA levels of the different isoforms of CD44 after CELF2 overexpression. Compared with the control group, the expressions of CD44s were significantly decreased after overexpression of CELF2, while the expression levels of five of nine other spliceosomes (CD44v4, v5, v7, v9, and v10), but not of CD44v6, were increased. In addition, negative correlation between CD44s and CELF2 mRNA was observed in pancreaic cancer tissues (Additional file 1: Figure S1D). The results were consistent with the results of our analysis of CD44 protein isoforms (Fig. 6E and F). Therefore, we speculated that CELF2 might cause the transition from CD44s to CD44v.

### CD44 silencing can suppress aggressive characteristics in pancreatic cancer

**Fig. 7:**
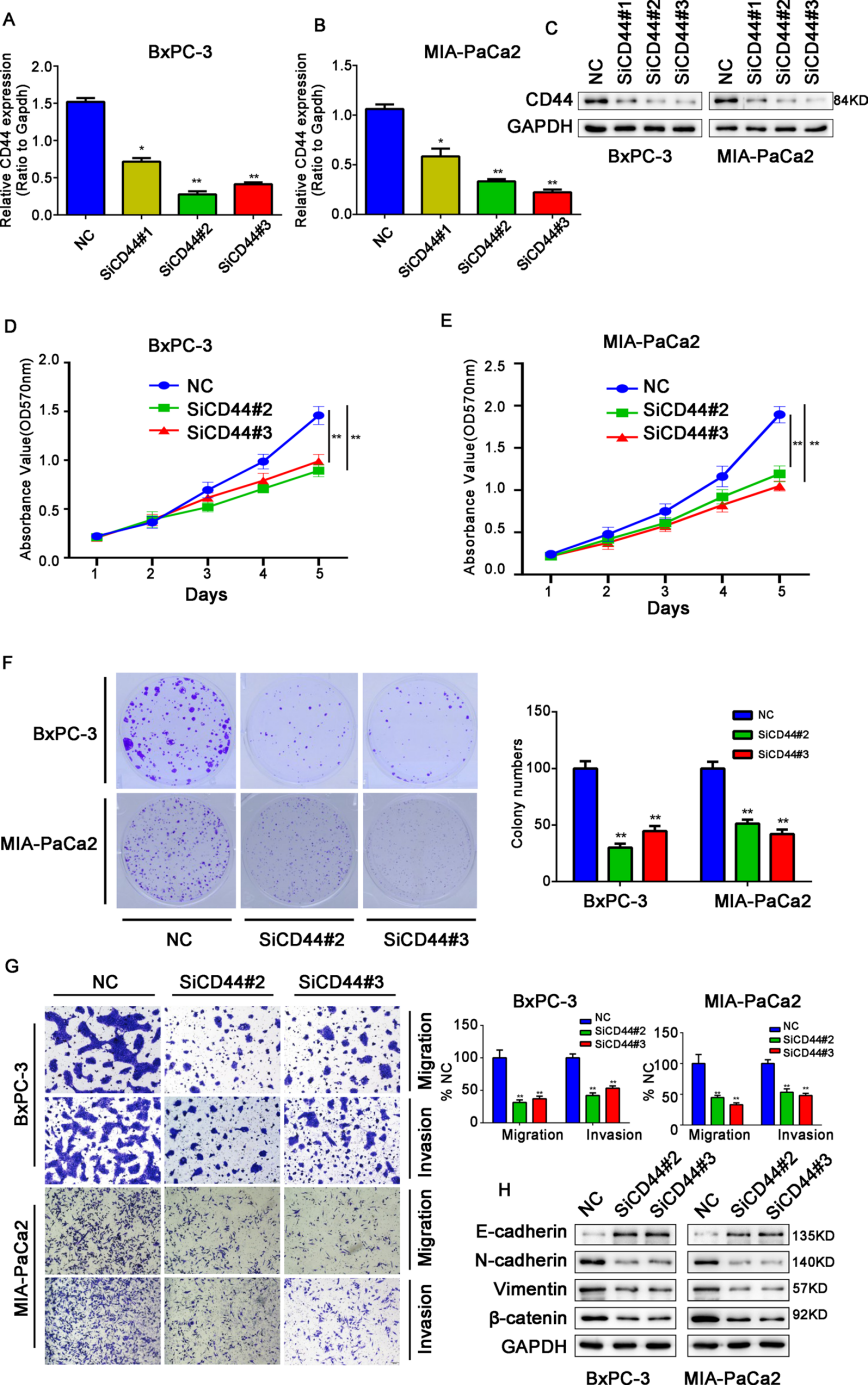
CD44 silencing inhibited tumorigenesis of PC, in agreement with the changes observed after CELF2 overexpression. **A**, **B** The effects of CD44 on pancreatic cancer cells were analyzed by transfecting BxPC-3 and MIA PaCa2 cells with different siRNAs (siCD44#1, siCD44#2, and siCD44#3). **C** Western blot was used to analyze the protein level of CD44 after the knockdown of CD44 in BxPC-3 and MIA Paca2 cell. **D**, **E** CCK-8 assay. **F** Plate clone formation assay. **G** Transwell assay. **H** EMT markers in CD44 knockdown cells were detected by western blot. Data are shown as the mean ± SD of three replicates. *P < 0.05, **P < 0.01

We further explored the effects of CD44 on PC cells by transfecting siRNAs (siCD44#2 and siCD44#3) into BxPC-3 and MIA PaCa2 cells (Fig. 7A–C). The CCK-8 assay showed that the proliferation ability of PC cells was significantly reduced after CD44 silencing compared with the control group (Fig. 7D and E). Compared with the NC group, the cloning ability of BxPC-3 and MIA PaCa2 cells silencing CD44 were significantly reduced (Fig. 7F). The Transwell assay showed that the invasion and migration ability of BxPC-3 and MIA PaCa2 cells was significantly reduced after CD44 silencing (Fig. 7G). To further reveal the effect of CELF2 regulating CD44 on PC, CELF2 and CD44 overexpression plasmids were co-transfected into PC cells, and it was observed that even CELF2 overexpression could not rescue the effect of CD44 overexpression on the proliferation, migration and invasion of the pancreatic cancer cells (Additional file 2: Figure S2), these results clearly indicated that CELF2 exerts its effects by modulating the expression of CD44. We further measured the expression levels of EMT-related proteins by western blot. The results showed that the level of the epithelial marker E-cadherin was increased, and the levels of the mesenchymal markers N-cadherin, vimentin, and β-catenin were significantly decreased in the siCD44 group compared with the control group (Fig. 7H). These results were consistent with the observed changes in the expression levels of EMT-related proteins in PC cells after overexpression of CELF2, and further confirmed that CELF2 might affect the biological behavior of PC cells by regulating the AS of CD44.

### CD44 silencing reduces PC cell stemness and promotes apoptosis of pancreatic cancer cells

**Fig. 8:**
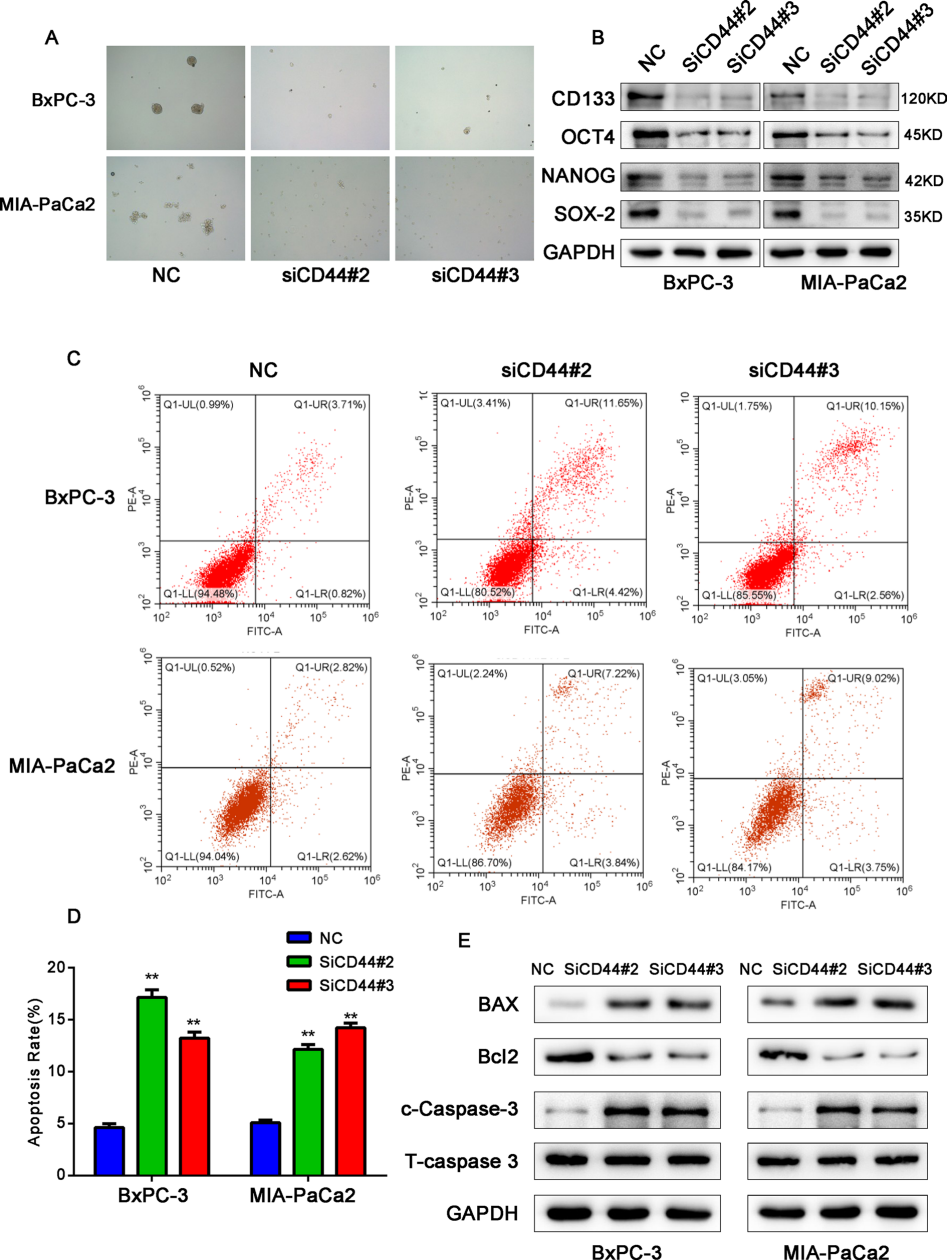
CD44 silencing reduced PC cell stemness and promoted apoptosis of pancreatic cancer cells. **A** and **B** CD44 maintains the stemness of PC cells. **A** Representative images of sphere formation induced by the transfection of CD44 siRNA into BxPC-3 and MIA PaCa2 cells. **B** The expression levels of PC cell stemness markers, including CD133, OCT4, NANOG, and SOX-2, were examined by western blot. **C** Flow cytometry was used to assess the apoptosis of PC cells in siCD44 and NC groups. **D** Statistical analysis of the effects of CD44 silencing on apoptosis. **E** The expression levels of apoptosis markers, including BAX, BCL-2, and caspase-3, were examined by western blot. Data are shown as the mean ± SD of three replicates. *P < 0.05, **P < 0.01

Sphere formation assays were conducted to further explore whether CD44 affects the cell stemness of PC cells. We found that the spheroidizing ability of PC cells was significantly inhibited when CD44 was silenced in BxPC-3 and MIA PaCa2 cells (Fig. 8A). The expression levels of the stem cell-like cell markers CD133, OCT4, NANOG, and SOX-2 in PC cells were measured by western blot. We found that the expression levels of these stem cell-like cell markers were significantly downregulated after CD44 was silenced compared with the control group (Fig. 8B). This was also consistent with the observed changes in the expression levels of stem cell-like markers in PC cells after overexpression of CELF2.

Then, we determined the effects of CD44 on apoptosis by flow cytometry. It was found that apoptosis was promoted after silencing of CD44 (Fig. 8C and D), in agreement with the observed changes after CELF2 overexpression. The expression of apoptosis-related proteins was measured by western blot. Compared with the control group, the expression of BCL-2 in PC cells was significantly decreased after silencing of CD44, and the expressions of caspase-3 and BAX were upregulated (Fig. 8E).

### CD44 affects the biological behavior of pancreatic cancer through regulation of endoplasmic reticulum-associated degradation

**Fig. 9:**
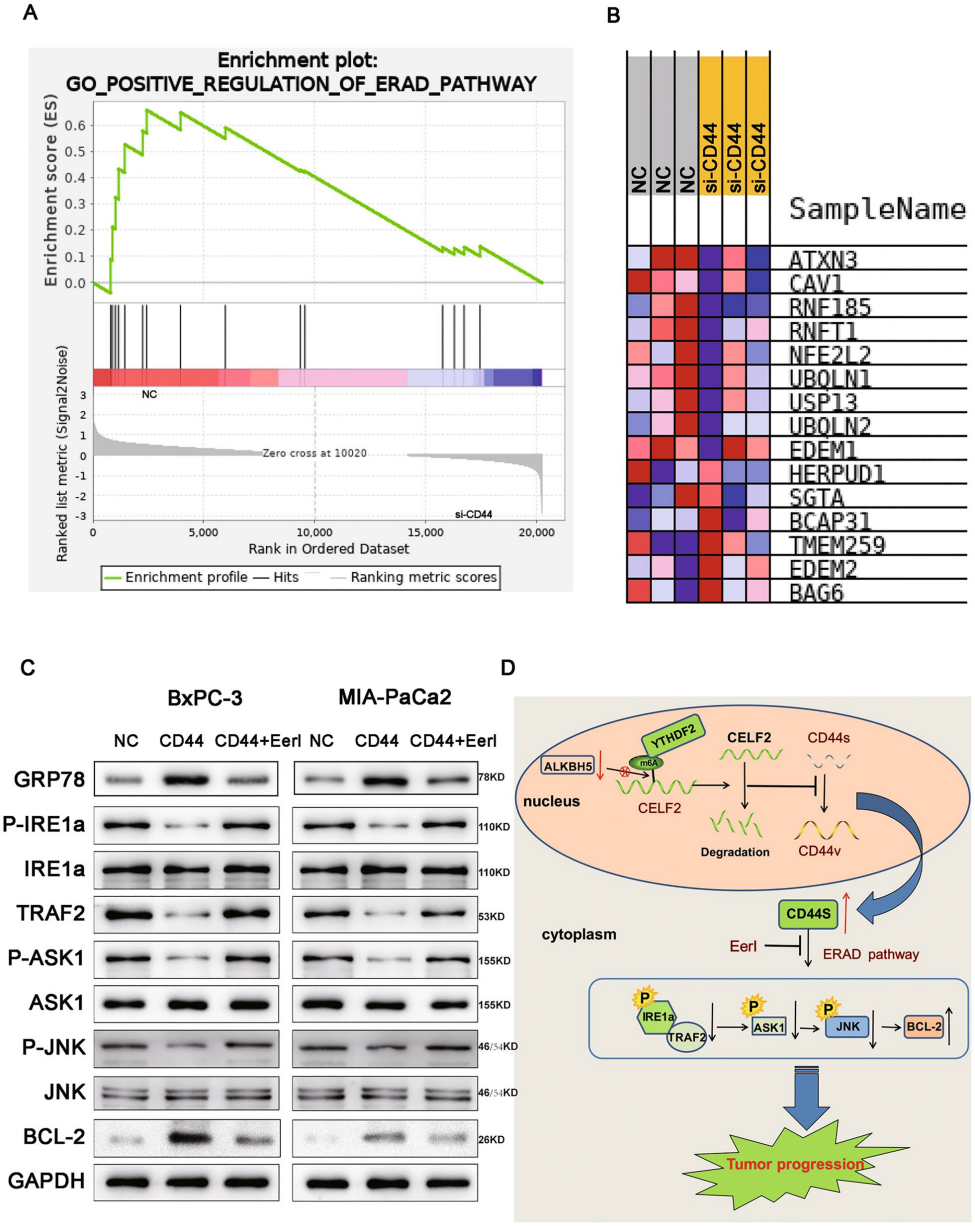
CD44 affects the biological behavior of PC through regulation of the endoplasmic reticulum degradation pathway.** A** GSEA of differentially expressed genes indicated that when CD44 was inhibited, the ERAD pathway was downregulated. **B** Heatmap showed differential genes enriched in ERAD signal pathway after silencing CD44. **C** ERS-related protein GRP78, IRE1a/p*-*IRE1a, TRAF2, ASK1/p-ASK1, JNK/p-JNK, BCL-2 were detected by Western-blot. **D** Schematic diagram of the proposed role of CELF2 in PC and its mechanism. ALKBH5-mediated m6A methylation unstabilizes and downregulates CELF2. CELF2 exerts oncogenic effect in PC by regulating CD44 alternative splicing to hinder CD44s switching towards CD44v, further inhibits p-IRE1a on the ER surface to form a dimer with TRAF2 to hinder the initiation of the mitochondrial apoptotic pathway, thus contributing to tumor progression of PC. Data are shown as the mean ± SD of three replicates. *P < 0.05, **P < 0.01

Transcriptomics analysis was conducted to identify differentially expressed genes after silencing of CD44. In total, 15,648 genes were differentially expressed (Additional file 3: Figure S3A), but these differences were significant for only 626 genes (P < 0.05) (Additional file 3: Figure S3B). Of these 626 genes, the 192 genes with |log_2_(fold change)|> 1 were selected for further analysis (75 upregulated and 117 downregulated genes) (Figure. S3C). GO functional enrichment analysis of these genes revealed that they were mainly enriched in the following GO terms: response to stress, cell proliferation, cell adhesion, cell motility, and regulation of biological process (Figure. S3D). KEGG pathway enrichment analysis revealed that differentially expressed genes were mainly enriched in endoplasmic reticulum (ER)-associated degradation (ERAD) signaling pathway proteins (Figure. S3E), and GSEA of differentially expressed genes revealed that when CD44 was inhibited, the ERAD pathway was inhibited (Fig. 9A, B). Therefore, we speculated that CD44 might affect the progression of PC through the ERAD signaling pathway. We further verified our hypothesis through follow-up experiments. Combined with the results of the above high throughput data analysis, ER is a vital cellular organelle which affect cell death, GO analysis showed that the differential genes were closely related to cell apoptosis and survival. As we known, after phosphorylation of IRE1a on the ER surface, it formed a dimer with TRAF2, which activated downstream ASK1 phosphorylation and further activated JNK1, thereby inducing mitochondrial apoptosis. Our results indicated that while CD44 was overexpressed, the phosphorylation level of IRE1a and TRAF2 would be significantly down-regulated, thereby inhibiting the subsequent phosphorylation of JNK1 and ASK1, hindering the initiation of the mitochondrial apoptotic pathway, and treatment of cells with ERAD pathway inhibitor EerI could reverse the effect of CD44 on ERAD pathway (Fig. 9C).

EerI is a small molecule that can significantly inhibit the ERAD pathway by inhibiting P97, an important molecule in said pathway. We treated cells with EerI to further explore the effects of CD44 on the ERAD signaling pathway. First, BxPC-3 and MIA PaCa2 cells were treated with different concentrations of EerI (Additional file 4: Figure S4A). As the concentration of EerI increased, the proliferation and spheronization ability of cells decreased significantly (Additional file 4: Figure S4B and C).

For subsequent experiments, an EerI concentration of 10 μM was selected. After treating BxPC-3 and MIA PaCa2 cells overexpressing CD44 with EerI, we found that EerI could reverse the effect of CD44 on PC, which significantly reduced tumor proliferation and promoted cell apoptosis (Additional file 5: Figure S5A and C). Not only that, we also found that the spheroidizing ability of PC cells was significantly inhibited by EerI in BxPC-3 and MIA PaCa2 cells (Additional file 5: Figure S5D), and the expression levels of stem cell-like cell markers CD133, OCT4, NANOG, and SOX-2 were significantly downregulated while treated with EerI (Additional file 5: Figure S5E). We further examined the expression level of GRP78, one of the main proteins involved in ER stress (ERS), and we found that after siliencing CD44, GRP78 was downregulated (Additional file 5: Figure S5F). The above results further confirmed that CD44 promotes ERS by activating the ERAD signaling pathway, thereby affecting the biological behavior of PC.

## Discussion

PC is a malignant tumor with a high mortality rate. The characteristics of insidious disease, strong invasiveness, and rapid disease progression make most patients lose the opportunity for surgery before the time of diagnosis. Therefore, research of the biological characteristics of PC cells may aid in the development of new treatment strategies for patients with advanced PC. In this work, we mainly focused on the role of variable splicing of genes in the occurrence and development of PC.

Splicing factors influence the effects of target genes by shearing target genes, thereby affecting the biological behavior of cancer cells. Studies have found that abnormal expression of splicing factors is involved in the progression of PC [15]. Therefore, the regulation of splicing factors may become a key means in regulating tumor cell activity and a potential target for the treatment of PC. Our study discovered a very interesting splicing factor, CELF2, which is significantly downregulated in PC tissue compared with pericarcinomatous tissue. Its low expression promotes the proliferation, invasion, and migration of tumors and plays a role in maintaining cell stemness. In addition, patients with lower CELF2 expression had shorter survival times. Studies confirmed that upregulation of CELF2 is associated with a stronger response of PC cells to chemotherapy. However, why it is downregulated and how it affects PC remains unclear. Therefore, we explored the mechanisms underlying the effects of CELF2 in PC.

In this study, we found that the main cause of low CELF2 expression in PC was CELF2 mRNA m6A modification by ALKBH5. The m6A modification has been proved to affect the level of gene methylation and regulate the stability of mRNA. We initially clarified the positive correlation between CELF2 levels and the demethylase ALKBH5. In subsequent experiments, we found that CELF2 could be demethylated by ALKBH5. The low expression of ALKBH5 induced high m6A level of CELF2 in PC cells, thus, m6A of CELF2 was read by YTHDF2, resulting in reduced stability of CELF2 mRNA, which further downregulated CELF2 expression. This theory could fully explain the low expression of CELF2 in PC. The above studies have fully confirmed that CELF2 may be a key gene regulating the occurrence and development of PC. Previous studies have demonstrated that CELF2 could play an important role in many kinds of tumors, including liver cancer [16], ovarian cancer [17], breast cancer [18], and gastric cancer [19]. Jakstaite et al. found that overexpression of CELF2 can enhance the chemosensitivity of PC cells [9]. CELF2 has been found to mediate curcumin-induced mitotic catastrophe of PC cells [20]. The role of CELF2 as a splicing factor has not yet been elucidated. We identified the AS events caused by CELF2 in PC based on the TCGA public database, and CD44 was its key targeted splicing gene. To our knowledge, this is the first study to explore the mechanism of CELF2 targeting AS of the gene encoding CD44, affecting the biological behavior of PC.

Different spliceosomes of CD44 play distinctive roles in various tumors. Some studies have shown that the conversion of CD44v to CD44s promotes tumor progression [21, 22]. However, other studies showed that CD44v plays a tumor-promoting role. When CD44s was conversed to CD44v, the tumors further progressed [23, 24]. The role of differently spliced isoforms of CD44 in PC is controversial [25] ^[26–28]^. In this study, we found that CD44s plays an important role in promoting tumor progression. After overexpression of CELF2, the expression of CD44s was significantly decreased by AS of the CD44 pre-mRNA, and different key spliceosomes were formed, which inhibited tumor progression. It is worth noting that the expression of CD44v6 also showed a downward trend when CD44s were downregulated. This indicated that CD44v6 may form the key spliceosome that affects the characteristics of PC. These results are consistent with the research of Zöller et al. [29]. To assess the roles of all CD44 spliceosomes in tumors, we knocked out CD44 in PC in the following experiments. Our results indicated that CD44 is also involved in PC progression. Regulation of AS of CD44 might be a novel treatment strategy for PC. Studies have shown that steric-blocking oligonucleotides can redirect AS. Moreover, the ability of RNA-blocking oligonucleotides to restore gene function makes them best suited for the treatment of genetic disorders [30]. At present, the antisense oligonucleotides method has been used in clinical trials for other diseases caused by AS, and whether it can be used in the treatment of PC remains to be further studied.

Finally, we explored how CELF2 affects PC after splicing CD44. Transcriptomics analysis revealed that on CD44 knockdown, the differentially expressed genes were mainly enriched in the ERAD pathway. The ER mainly regulates the synthesis, processing, and degradation of proteins. Tumor cells can eliminate misfolded proteins through ERS and the ubiquitin protease pathway to maintain cell survival. Therefore, inhibition of the ERAD pathway may promote tumor cell apoptosis and inhibit tumor growth. Studies have shown that abnormal activity of the ERAD pathway plays an important role in tumor development [31, 32]. EerI is an inhibitor of p97, an important molecule in the ERAD pathway, which can significantly inhibit the ERAD pathway. EerI inhibits the proliferation and promotes apoptosis of PC cells [33], but the underlying mechanism is not clear. In this study, EerI significantly reversed the activation effect of CD44 on ERAD, which further confirmed that CD44 could promote the occurrence and development of PC through the activation of ERAD. To our knowledge, we are the first to propose that CELF2-mediated CD44 AS affects PC through the ERAD pathway.

## Conclusions

In summary, this study indicates that N6-methyladenosine-mediated CELF2 promotes AS of CD44, affecting the ERAD pathway and regulating the biological behavior of PC cells. We also found that EerI reversed the positive effects of CD44 on PC and provided a new strategy for the development of anti-tumor drugs. CELF2 is expected to be a new target for targeted-drug development.

## Methods

### Ethics statement and subjects

In total, 80 patients diagnosed with PC who were operated in the First Affiliated Hospital of Fujian Medical University between 2009 and 2020 were included in this study. The clinicopathological characteristics including age, sex, alcohol history, smoking history, CEA, CA199, tumor size, tumor stage, lymph node invasion, and metastasis were recorded. The current study was approved by the Ethics Committee of the First Affiliated Hospital of Fujian Medical University (Fujian, China).

### Cell lines and cell culture conditions

All of the cell lines used in this study were purchased from the American Type Culture Collection (ATCC; Manassas, VA, USA), including the cancerous AsPC-1, CFPAC-1, Capan-1, SW1990, PANC-1, SW1990, BxPC-3, and MIA-PaCa-2 cell lines and the non-cancerous HPDE6C7 cell line. All of the above-mentioned cells were cultured in 5% CO_2_ at 37 °C in 100% humidity in Dulbecco’s modified Eagle’s medium (DMEM) containing 10% fetal bovine serum (FBS) and 1% antibiotic/antifungal solution (Biowest, Nuaillé, France).

### Immunohistochemistry

All of the tissues were fixed with 10% formalin for at least 48 h and paraffin-embedded for immunohistochemistry (IHC) assays, as described previously [10]. We incubated tissue sections with primary antibodies (Table 1) at 4 °C overnight and treated them with an UltraVision Quanto Detection System Horseradish Peroxidase (HRP) 3,3′-Diaminobenzidine (DAB) Kit (Thermo Scientific [Thermo Fisher Scientific, Waltham, MA, USA]) as per the manufacturer’s instructions. Expression changes were identified as follows: 10 highly magnified fields were randomly selected for counting under an Olympus CX23 microscope (Olympus Corp., Tokyo, Japan) at 200 × resolution, and cancer cells were counted under the microscope as previously described [10].

### Western blot

Total protein was extracted from homogenized tissues or cells, and the lysate was mixed with hydrolysis buffer. We determined the protein concentration using the bicinchoninic acid (BCA) method. Bromophenol blue and β-mercaptoethanol were added into the sample buffer before electrophoresis. After separation by 10% sodium dodecyl sulfate polyacrylamide gel electrophoresis (SDS-PAGE), the proteins were transferred to a polyvinylidene fluoride membrane (Bio-Rad Laboratories, Hercules, CA, USA). The membrane was incubated with primary antibody (Table S1) at 4 °C overnight. After incubation for 2 h in secondary antibody, the protein bands were visualized using enhanced chemiluminescence (ECL) substrate and photographed. ImageJ software version 1.8.0 (National Institutes of Health, Bethesda, MD, USA) was used to examine relative expression of proteins. Each experiment was repeated in triplicate.

### Quantitative reverse-transcriptase PCR

Total RNA was extracted from selected cells or tissues using TRIzol reagent (Invitrogen, Carlsbad, CA, USA) as per the manufacturer’s instructions. We examined the RNA integrity and purity using agarose gel electrophoresis. [11]. Total reverse-transcription primers (Table S2), and ImPro-II reverse transcriptase (Promega, Fitchburg, WI, USA) were used for reverse transcription per the manufacturer’s instructions. For quantitative reverse-transcriptase PCR (qRT-PCR), we used Fast Start Universal SYBR Green Master Mix (Roche Diagnostics GmbH, Mannheim, Germany).

### Colony formation and holoclone sphere formation assays

For the colony formation assay, first, the cells were cultured for 14 days in 6-well plates at 5 × 10^2^ cells per well. Then, the cells were fixed by methanol/acetic acid (3:1, v/v) and stained with 0.5% crystal violet (Sigma-Aldrich, St. Louis, MO, USA). The resulting cells were counted using a microscope (Olympus IX81).

For the holoclone assay, 100 cells were cultured for several days in a 6-well dish, and then the number of holoclones was counted. Cloning efficiency was defined as the percentage of cells that established a holoclone.

### Flow cytometry

The cells were suspended in 1 × 10^6^ cells/ml, and 5 μL Annexin V and propidium iodide (PI) staining solution were added to 300 μL of the cell suspension. Stained cells were assayed by a FACSort Flow Cytometer (BD, San Jose, CA, USA) after incubation at room temperature for 10–15 min.

### Cell counting kit-8 (CCK-8)

The cells were seeded in 96-well plates with 1 × 10 3 cells/well, and unceasingly cultured at 37° C for 0∼96 h. Subsequently, 10 μL CCK-8 reaction reagent (Dojindo, Japan) was respectively added to each well and incubated at 37° C for 2 h, the absorbance value at 450 nm was detected on a microplate reader (spectra-max plus384, Molecular Devices, USA).

### RNA immunoprecipitation assay

A Magna RIP™ RNA-Binding Protein Immunoprecipitation Kit (Millipore, USA) was used for the RNA immunoprecipitation (RIP) assay according to the manufacturer’s instructions. Briefly, cell extracts were immunoprecipitated with Sepharose bead-conjugated antibodies against m6A, YTHDF2 or IgG at 4 °C for 6 h. To remove proteins from the complex, 0.1% SDS/Proteinase K (0.5 mg/mL, 30 min at 55 °C) was used. Immunoprecipitated proteins and RNAs were detected using western blot and qRT-PCR, respectively.

### Transcriptome sequencing

Total RNA was isolated and purified using TRIzol (Life, cat. 265709, CA, USA) following the manufacturer’s procedure. After inspecting the quality of the total RNA using an Agilent 2100 Bioanalyzer (Agilent, cat. G2939AA, CA, USA) and a NanoPhotometer® (Implen, cat. N60, Munich, Germany), mRNA with poly(A) was purified from 1 μg total RNA using VAHTS® mRNA Capture Beads with Oligo(dT) (Vazyme, cat. N401-01, Nanjing, China) through two rounds of purification. Subsequently, mRNA fragments were interrupted using the VAHTS® Universal V6 RNA-seq Library Prep Kit (Vazyme, cat. NR604, Nanjing, China) at 94 °C for 8 min and reversed transcribed into cDNA, which was used to synthesize U-labeled double-stranded DNA. An A-base was added to the blunt ends of each strand to ligate the indexed adapters which contain a T-base at the tail end. After UDG enzyme treatment of the U-labeled double-stranded DNA, size selection was performed with VAHTS® DNA Clean Beads (Vazyme, cat. N411, Nanjing, China). Then the ligated products were amplified with PCR under the following conditions: initial denaturation at 98 °C for 5 min; 12–17 cycles of denaturation at 98 °C for 10 s, annealing at 60 °C for 30 s, and extension at 72 °C for 30 s; final extension at 72 °C for 5 min. The average insert size of the cDNA library was 280 ± 80 bp. After purification by VAHTS® DNA Clean Beads (Vazyme, cat. N411-02, Nanjing, China), quality control of concentration and fragment size was performed using an Agilent 2100 Bioanalyzer (Agilent, cat. G2939AA, CA, USA) and Qubit assay tubes (Life, cat. 1604220, CA, USA). At last, we performed the 2 × 150-bp paired-end sequencing (PE150) on an Illumina NovaSeq™ 6000 system (Illumina Corporation, San Diego, USA) following the vendor’s recommended protocol by Guangzhou Huayin Health Medical Group CO., Ltd. (Guangzhou, China). The mRNA expression levels were calculated using RNA-Seq by Expectation Maximization (RSEM) (v1.3.1) and normalized to fragments per kilobase per million reads (FPKM) values. The differentially expressed mRNAs were screened using the edgeR package (https://bioconductor.org/packages/release/bioc/html/edgeR.html) in R. Genes with |log_2_(fold change)|≥ 1, and P < 0.05 was considered to be significantly differentially expressed. Subsequently, GO and KEGG pathway enrichment analyses were conducted using the clusterprofiler package in R. GSEA_Linux_4.0.3 (https://www.gsea-msigdb.org/gsea/index.jsp) was used to complete the enrichment analysis of the gene set with default parameters using the expression file obtained by RSEM as input.

### Overexpression plasmid and siRNA transfection

We purchased the overexpression plasmid and negative controls (NCs) of CELF2 from GenePharma (Shanghai, China). Cells were seeded into 6-well plates at a concentration of 5 × 10^5^ cells/2 ml per well. After 48 h, cells reached 60% of confluence. We then transfected the cells with overexpression plasmid or NCs using Lipofectamine 3000 (Invitrogen) as per the manufacturer’s instructions at a final concentration of 50 nM. Small interfering RNAs (siRNAs) targeting CD44 were transfected into cells using the methods described above (Table S3). The final concentration of siRNA was 100 nM.

### Cell migration and invasion assay

To detect cell migration and cell invasion, we used Transwell chambers (pore size, 8 μm; BD Biosciences, Franklin Lakes, NJ, USA). These chambers were coated with Matrigel (Corning Labware Products Inc., Corning, NY, USA) for cell invasion assays, and they were left uncoated for cell migration assays. For both types of assays, we seeded PC cells into the upper chamber of the Transwell and added medium containing serum to the bottom chamber. After 24 h, cells that had migrated into the Matrigel coating layer were fixed with paraformaldehyde and then stained with crystal violet to allow visualization. We observed, counted, and photographed the cells under an Olympus IX73 microscope (Olympus).

### Lentivirus transduction

The lentivirus pEZX-MR04 plasmid expressing CELF2 and NCs were obtained from GeneCopoeia Inc. (Rockville, MD, USA). EndoFectin Lenti transfection reagent was used to co-transfect lentiviruses expressing CELF2 or the NC into HEK293T cells. After 48 h incubation, lentiviral particles were harvested by centrifugation (500 g for 10 min) and filtered. Cells were transduced using the lentivirus expressing CELF2 or NC. Stably transduced cells were selected for further study. Cells were cultured for 14 days in the presence of puromycin (2 μg/ml). The gene expression levels of CELF2 were measured using qRT-PCR.

### In vivo assay to determine tumor growth

Male BALB/c nude mice (average age, 6 weeks) from the Shanghai Laboratory Animal Center were acquired for mouse experiments. All of the mouse protocols were reviewed and approved by the Fujian Medical University Ethics Review Committee. Cells stably transfected with the CELF2 overexpression lentivirus or empty vectors were subcutaneously injected into the hypochondriac regions of the selected mice. Tumor formation in these mice was observed for 5 weeks. Then the tumors were harvested for weight and volume measurements.

### Statistical analysis

SPSS software version 21.0 (IBM Corp., Armonk, NY, USA) was used for statistical analysis. All of the data are reported as mean ± standard deviation (SD). Data were evaluated using Student’s t-test or one-way ANOVA. The χ^2^ test was used to evaluate correlations between expression levels and clinicopathological features. Pearson’s correlation coefficient was used for the correlation analysis. The Kaplan–Meier approach was used for survival analysis estimates. P < 0.05 was considered to be statistically significant.

**Table 1:** Relationship between CELF2 expression levels and clinicopathological parameters in 80 pancreatic cancer patients

Variables	All cases	CELF2 expression		*P*
		Low (*n* = 48)	High (*n* = 32)	
Age (years)				
<50	43	22	21	0.082
≥50	37	26	11	
Gender				
Male	51	32	19	0.506
Female	29	16	13	
Alcohol history				
No	64	38	26	0.819
Yes	16	10	6	
Smoking				
No	28	17	11	0.894
Yes	52	30	22	
CEA				
Normal		16	9	0.622
High		32	23	
CA199				
Normal		13	11	0.486
High		35	21	
Tumor size (cm)				
<4	38	18	20	0.028^*^
≥4	42	30	12	
TNM stage				
I–II	35	16	19	0.02
III–IV	45	32	13	
Lymph node invasion				
Absent	36	17	19	0.035^*^
Present	44	31	13	
Metastasis				
No	63	34	29	0.034
Yes	17	14	3	

*P < 0.05.

## Supplementary Information

**Additional file 1: Figure S1**. (A) The expression of ALKBH5 protein in pancreatic cancer tissues, precarcinomatous tissue and cell lines. (B) The expression of YTHDF2 protein in pancreatic cancer tissues, precarcinomatous tissue and cell lines. (C) The expression of CD44 isoforms in the PC control cell line HPDE6-C7. (D) Negative correlation between CD44s and CELF2 mRNA was observed in pancreaic cancer tissues.

**Additional file 2: Figure S2**. CELF2 overexpression could not rescue the effect of CD44 overexpression on the proliferation, migration and invasion of the pancreatic cancer cells. (A) The CCK-8 assay was presented the proliferation of the pancreatic cancer cells in the four grops. (B-C) Transwell analysis was presented the invasion and migration of BxPC-3 and MIA PaCa2 cells. Data are shown as the mean ± SD of three replicates. *P < 0.05, **P < 0.01.

**Additional file 3: Figure S3**. Differentially expressed genes after silencing CD44 were identified by transcriptomics analysis. Venn diagram displaying 15,648 genes whose expression levels were different between the siCD44 and negative control groups. (B) Volcano diagram showing the differentially expressed genes. (C) Histogram showing that of 192 significantly differentially expressed genes (|log2(fold change)| > 1, P < 0.05), 75 genes were upregulated and 117 genes were downregulated. (D) Functional enrichment analysis indicated that the differentially expressed genes were mainly enriched in the following GO terms: response to stress, cell proliferation, cell adhesion, cell motility, and regulation of biological process. (E) KEGG pathway enrichment analysis indicated that differentially expressed genes were mainly enriched in the ERAD signaling pathway.

**Additional file 4: Figure S4**. As the concentration of EerI increased, the proliferation and spheronization ability of cells decreased significantly. (A and B) Cell viability was determined in BxPC-3 and MIA PaCa2 cells treated with different concentrations of EerI. (C and D) CCK8 assay. (E) Representative images of sphere formation. Data are shown as the mean ± SD of three replicates. *P < 0.05, **P < 0.01.

**Additional file 5: Figure S5**. After treating BxPC-3 and MIA PaCa2 cells overexpressing CD44 with EerI, the effects of CD44 on PC were reversed. PC cell proliferation was analyzed by CCK-8 assay. (B) Apoptosis was examined by flow cytometry. (C) The expression of apoptosis-related proteins was assessed by western blot. (D) Representative images of sphere formation of BxPC-3 and MIA PaCa2 cells. The surviving colonies were measured for the number of tumorspheres. (E) The expression levels of stem cell-like cell markers CD133, OCT4, NANOG, and SOX-2 were detected by western-blot. (F) The expression levels of GRP78, one of the main proteins involved in endoplasmic reticulum stress (ERS), were analyzed by western blot. Data are shown as the mean ± SD of three replicates. *P < 0.05, **P < 0.01.

## Data Availability

All the data obtained in current study were available from the corresponding authors on reasonable request.

## Abbreviations

AS: Alternative splicing
PC: Pancreatic cancer
CUGBP: Cytidine-uridine-guanosine-binding protein 2
CELF2: CUGBP Elav-like family member 2
M6A: N6-methyladenosine
ERAD: Endoplasmic reticulum-associated degradation
ERS: Endoplasmic reticulum stress
IHC: Immunohistochemistry
FBS: Fetal bovine serum
RIP: RNA immunoprecipitation
