# Supplementary figures and images for: N6-methyladenosine-mediated CELF2 regulates CD44 alternative splicing affecting tumorigenesis via ERAD pathway in pancreatic cancer
Source: Cell Biosci. 2022 Aug 8;12:125. doi: 10.1186/s13578-022-00844-0 (PMC9361702; doi:10.1186/s13578-022-00844-0)

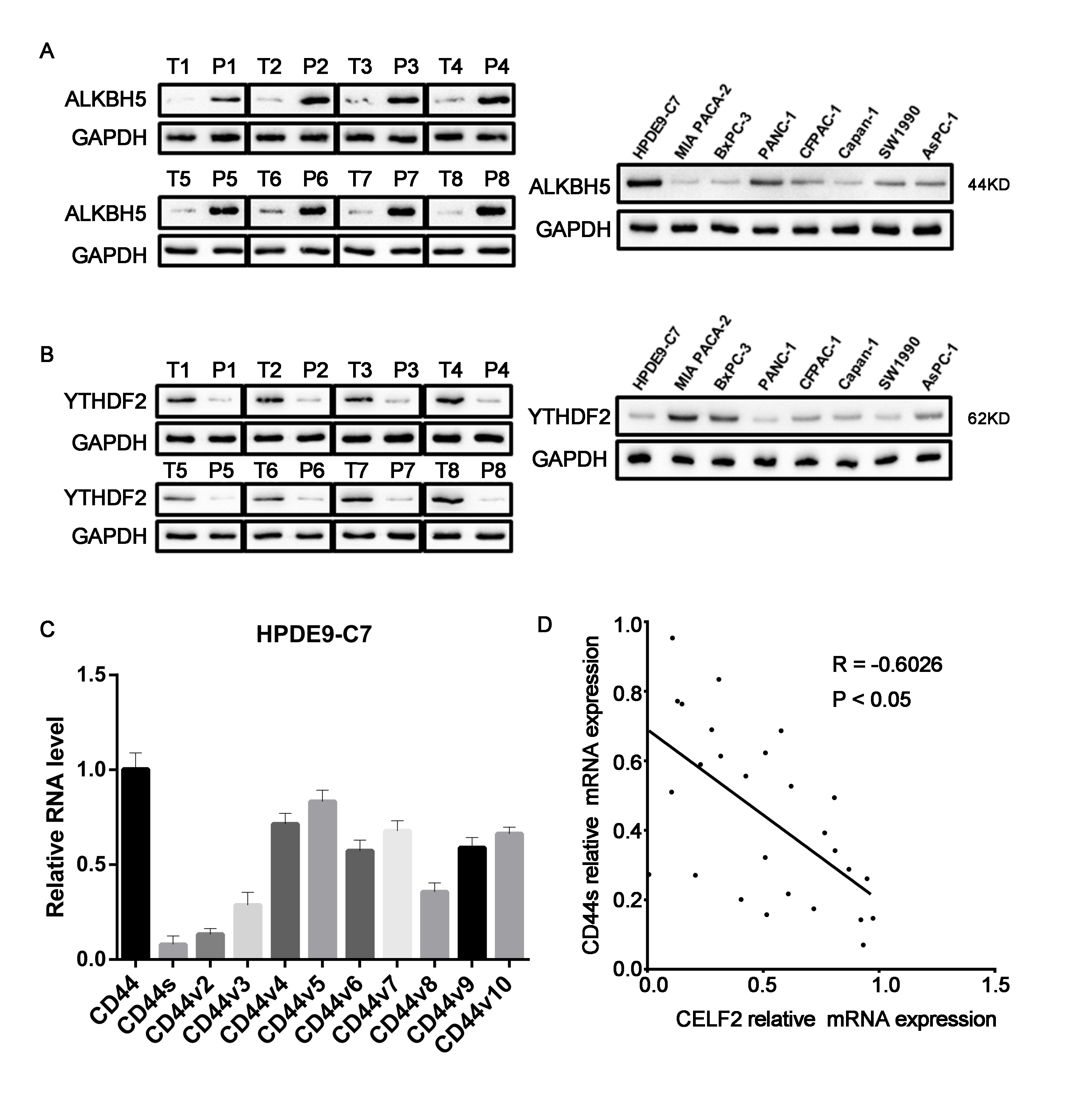

Supplement: Supplementary file 1 — Additional file 1: Figure S1. (A) The expression of ALKBH5 protein in pancreatic cancer tissues, precarcinomatous tissue and cell lines. (B) The expression of YTHDF2 protein in pancreatic cancer tissues, precarcinomatous tissue and cell lines. (C) The expression of CD44 isoforms in the PC control cell line HPDE6-C7. (D) Negative correlation between CD44s and CELF2 mRNA was observed in pancreaic cancer tissues. [file 13578_2022_844_MOESM1_ESM.tif]

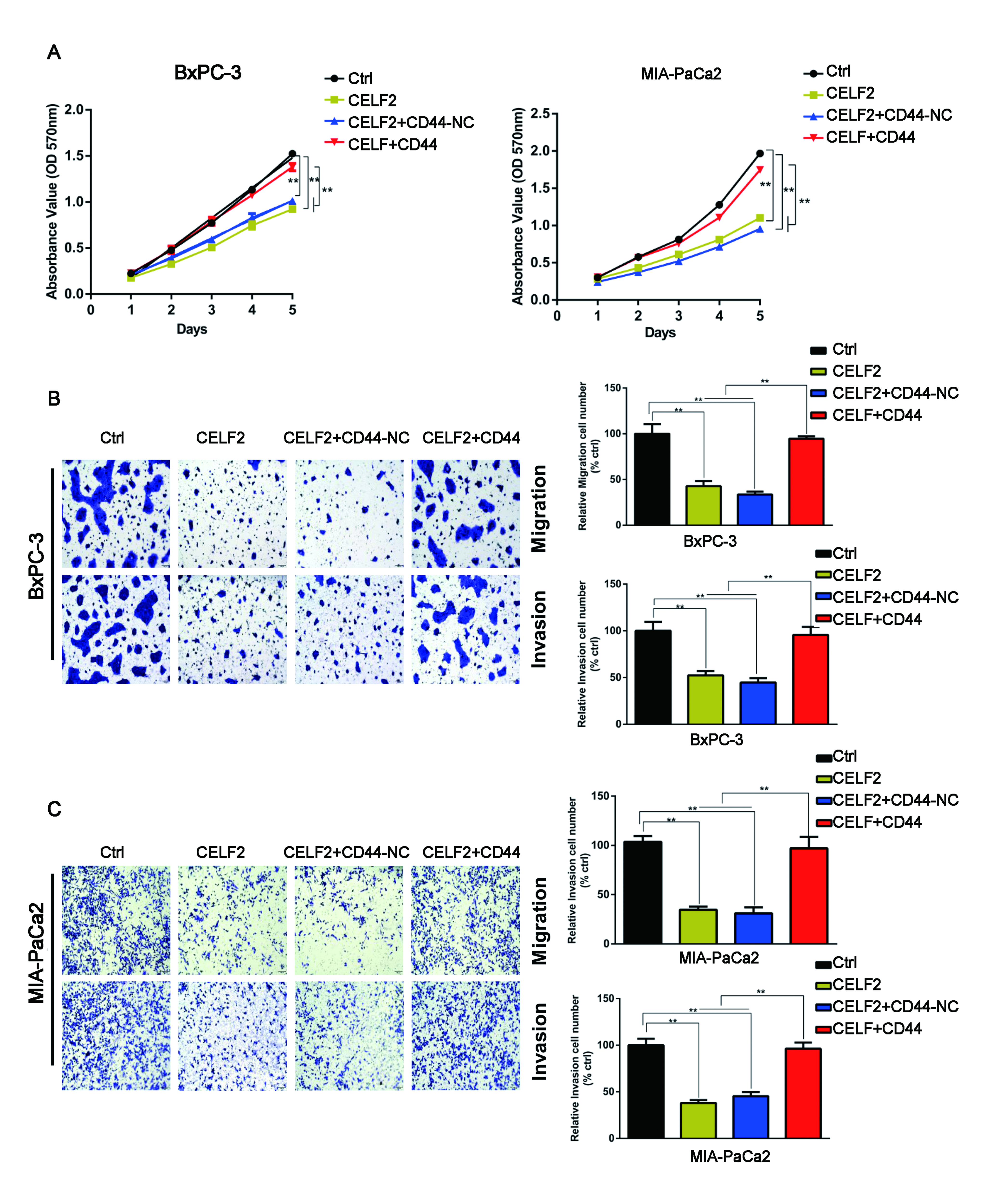

Supplement: Supplementary file 2 — Additional file 2: Figure S2. CELF2 overexpression could not rescue the effect of CD44 overexpression on the proliferation, migration and invasion of the pancreatic cancer cells. (A) The CCK-8 assay was presented the proliferation of the pancreatic cancer cells in the four grops. (B-C) Transwell analysis was presented the invasion and migration of BxPC-3 and MIA PaCa2 cells. Data are shown as the mean ± SD of three replicates. *P < 0.05, **P < 0.01. [file 13578_2022_844_MOESM2_ESM.tif]

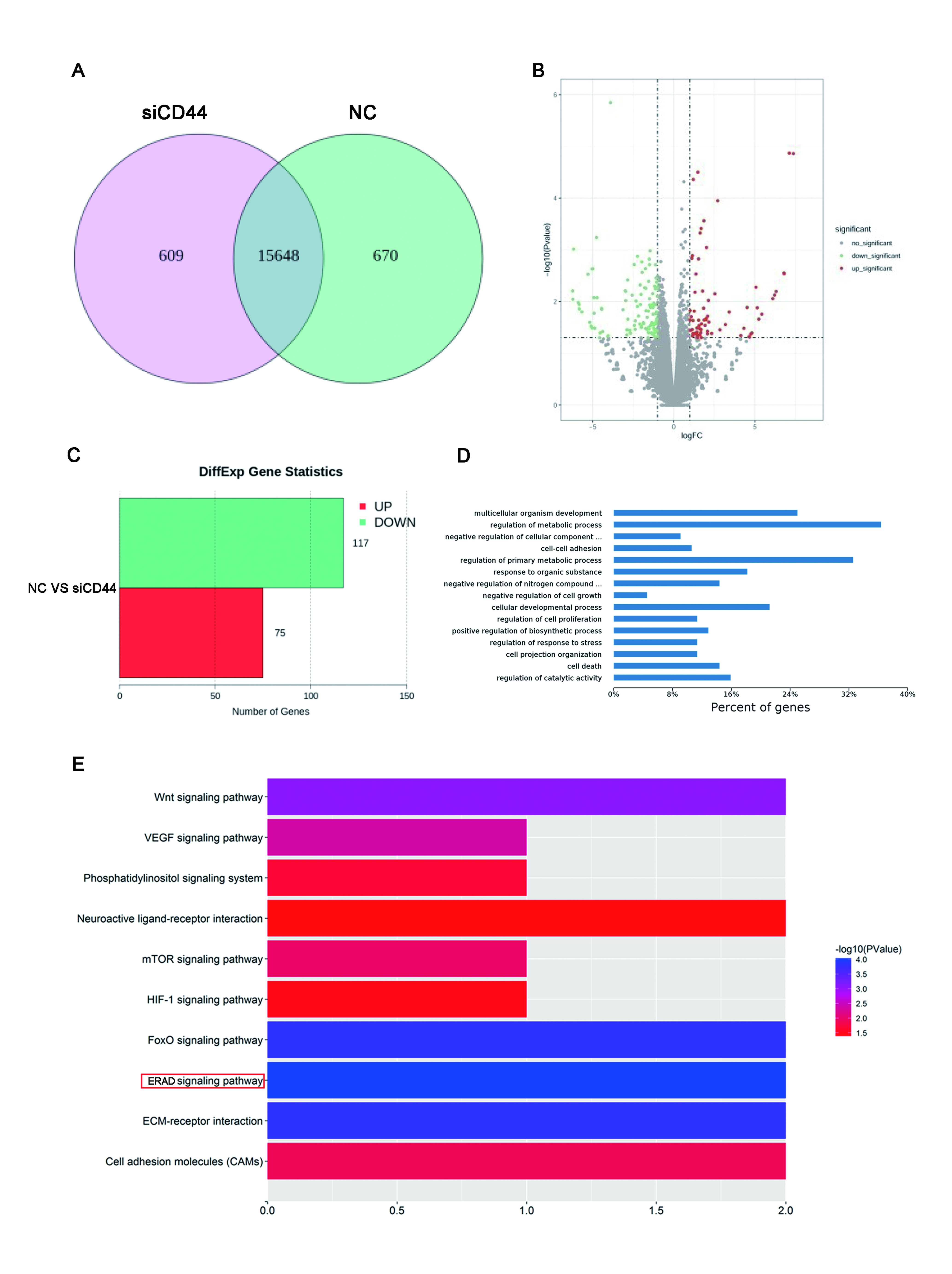

Supplement: Supplementary file 3 — Additional file 3: Figure S3. Differentially expressed genes after silencing CD44 were identified by transcriptomics analysis. Venn diagram displaying 15,648 genes whose expression levels were different between the siCD44 and negative control groups. (B) Volcano diagram showing the differentially expressed genes. (C) Histogram showing that of 192 significantly differentially expressed genes (|log2(fold change)| > 1, P < 0.05), 75 genes were upregulated and 117 genes were downregulated. (D) Functional enrichment analysis indicated that the differentially expressed genes were mainly enriched in the following GO terms: response to stress, cell proliferation, cell adhesion, cell motility, and regulation of biological process. (E) KEGG pathway enrichment analysis indicated that differentially expressed genes were mainly enriched in the ERAD signaling pathway. [file 13578_2022_844_MOESM3_ESM.tif]

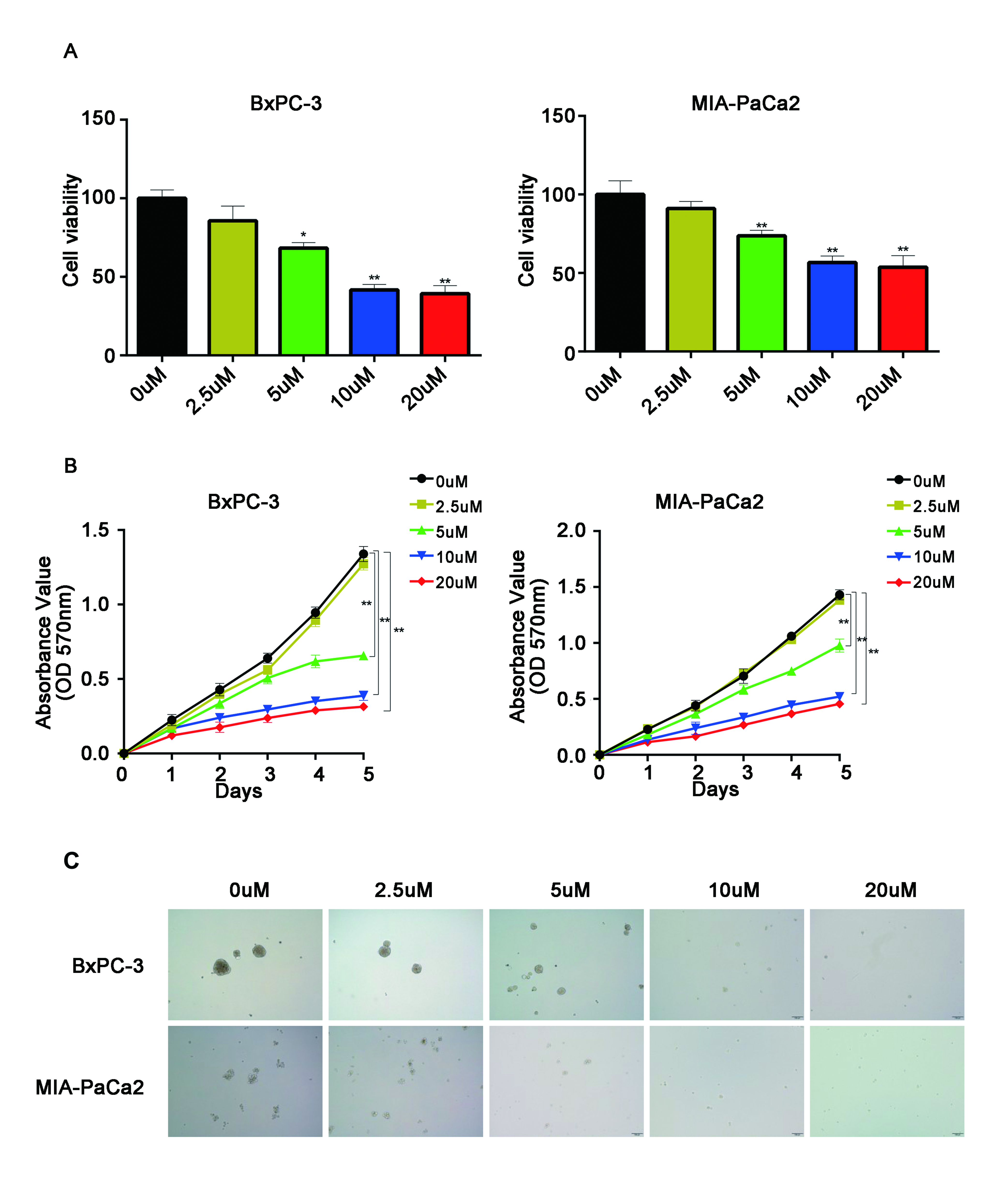

Supplement: Supplementary file 4 — Additional file 4: Figure S4. As the concentration of EerI increased, the proliferation and spheronization ability of cells decreased significantly. (A and B) Cell viability was determined in BxPC-3 and MIA PaCa2 cells treated with different concentrations of EerI. (C and D) CCK8 assay. (E) Representative images of sphere formation. Data are shown as the mean ± SD of three replicates. *P < 0.05, **P < 0.01. [file 13578_2022_844_MOESM4_ESM.tif]

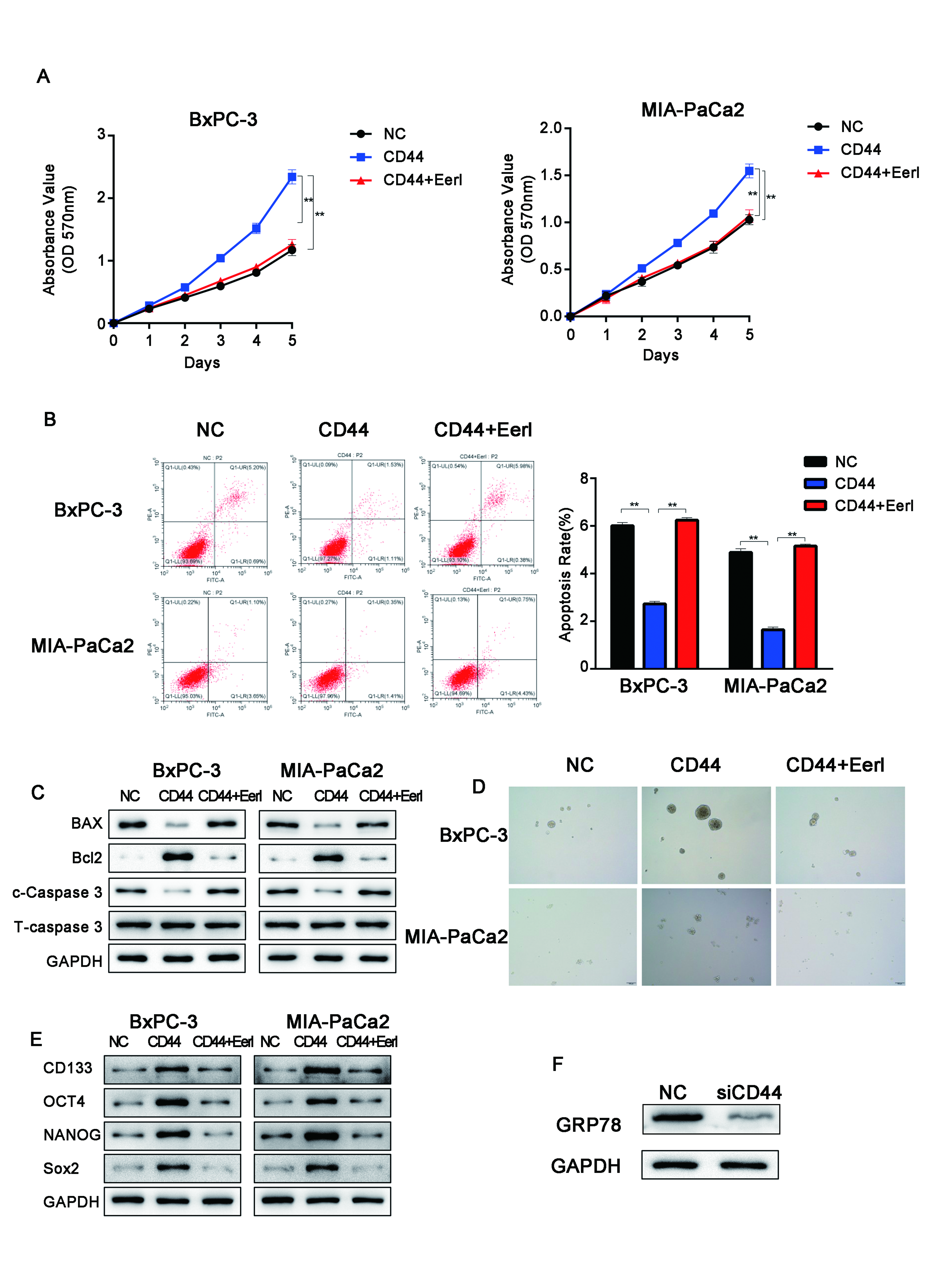

Supplement: Supplementary file 5 — Additional file 5: Figure S5. After treating BxPC-3 and MIA PaCa2 cells overexpressing CD44 with EerI, the effects of CD44 on PC were reversed. PC cell proliferation was analyzed by CCK-8 assay. (B) Apoptosis was examined by flow cytometry. (C) The expression of apoptosis-related proteins was assessed by western blot. (D) Representative images of sphere formation of BxPC-3 and MIA PaCa2 cells. The surviving colonies were measured for the number of tumorspheres. (E) The expression levels of stem cell-like cell markers CD133, OCT4, NANOG, and SOX-2 were detected by western-blot. (F) The expression levels of GRP78, one of the main proteins involved in endoplasmic reticulum stress (ERS), were analyzed by western blot. Data are shown as the mean ± SD of three replicates. *P < 0.05, **P < 0.01. [file 13578_2022_844_MOESM5_ESM.tif]
